# Metals, Health and the Environment – Emergence of Correlations Between Speciation and Effects

**DOI:** 10.1155/S1565363304000196

**Published:** 2004

**Authors:** David R. Williams

**Affiliations:** School of Chemistry Cardiff University P 0 Box 912 Cardiff CF10 3TB UK

## Abstract

Over the last half-century both the identification of the causes of diseases and the use of inorganic compounds to treat such conditions have been considerably enlightened through our emerging capabilities to identify the pivotal chemical species involved. The ‘duty of care’ placed upon scientists to protect the environment from manufactured chemicals and to limit their effects upon humans therefrom is best realised from a speciation knowledge database. This paper discusses categorising chemicals in terms of their persistence, bioaccumulation, and toxicities and uses speciation information to optimise desirable effects of chemicals in several applications such as the manufacture of pulp for paper and in the foliar nutrition of crops. Simultaneously, the chemical wasting side effects of industrial overdosing is easily avoided if speciation approaches are used. The move towards new environmentally friendly ligand agents is described and methods of finding substitute agents (often combinations of two or more chemicals) to replace nonbiodegradable EDTA. The geosphere migration of metals through the environment is discussed in terms of speciation. Future objectives discussed include improved means of communicating speciation-based recommendations to decision makers.


### Metals, Health and the Environment – Emergence of Correlations Between Speciation and Effects

David R Williams

*School of Chemistry, Cardiff University, P O Box 912, Cardiff CF10 3TB, UK, Tel/Fax 44 (0) 29 2087 4778, Williamsd@cardiff.ac.uk*

### ABSTRACT

Over the last half-century both the identification of the causes of diseases and the use of inorganic compounds to treat such conditions have been considerably enlightened through our emerging capabilities to identify the pivotal chemical species involved. The 'duty of care' placed upon scientists to protect the environment from manufactured chemicals and to limit their effects upon humans therefrom is best realised from a speciation knowledge database. This paper discusses categorising chemicals in terms of their persistence, bioaccumulation, and toxicities and uses speciation information to optimise desirable effects of chemicals in several applications such as the manufacture of pulp for paper and in the foliar nutrition of crops. Simultaneously, the chemical wasting side effects of industrial overdosing is easily avoided if speciation approaches are used. The move towards new environmentally friendly ligand agents is described and methods of finding substitute agents (often combinations of two or more chemicals) to replace non-biodegradable EDTA. The geosphere migration of metals through the environment is discussed in terms of speciation. Future objectives discussed include improved means of communicating speciation-based recommendations to decision makers.

### INTRODUCTION.

Multinational agencies such as the EU and the OECD are now focussing upon new means of chemical analysis which identify and reduce hazards to the health of their citizens and enables risk reduction management. Chemical speciation studies are one such promising approach /1/.

Notable successes have already been reported in linking environment with human health. From a new awareness which commenced with the publication of 'Silent Spring' in the 1960s, there has been increasing pressure to legislate and to enforce readily-bio-degradability criteria upon use of organic chemicals so that they rapidly disappear after use /2/. A more cautious approach is used with inorganic agents since the elements do not bio-degrade *per se* but do change their bonding, and sometimes oxidation states, to give another species /3/. The fact that such bonding exchanges are frequently labile in the environment, in humans, and in healthcare, has not been widely understood by non-chemistry scientists even though Sillén *et al* commenced this re-education process for geologists in the 1950-60s /4/.

The lability, inertness and speciation composition ground rules became encompassed into the emerging subject of bio-inorganic chemistry which considered the influence of metal-containing chemical species upon the environment and upon health /5,6/.

By the late 20th century strict criteria had been established (a) for the acceptance, or use subject to specific risk management standards, of organic ligands found in these complexes /7/. However, (b) the acceptability and disposal of non-biodegradable metals has yet to be fully defined and legislated.

This paper describes progress as seen from the viewpoint of chemicals in the UK which closely reflects the EU position, and shows how chemical speciation answers many current problems /8/.

### RESULTS

### (a) Speciation data for organic agents.

The challenge of such agents to the environment will be discussed in part (c ) under persistence, bio-accumulation, and toxicity (PBT) criteria /8/. Considering readily-biodegradability (the converse of persistence), this refers to aqueous reactions in the environment into which the used chemical is added. Typically this is in rivers and drains. Thus, the OECD-definitions of this term rely upon naturally occurring bacteria to degrade the organic agent within a specified time /7/.

This does not permit the intentional introduction of microbiologically selected bacteria having a specific affinity for the organic substrate. Further, it must be realised that even naturally occurring ground water bacteria require the prevailing conditions, such as temperature and light, *etc,* to be appropriate to their biodegrading activities. Thus, the readily biodegradable criteria embody terms such as the percentage of agent biodegraded within a certain time (typically 10 days) after the initiation period has ended. Further the measurement of the extent of degradation of a living system is a serious challenge. Some definitions use amount of carbon dioxide released, others use residual amount of organic matter remaining awaiting degradation (see Table 1).

This realisation that degradation is dependent upon commonly occurring bacteria suggests that including natural chemical groups within the formula of the manufactured agent will "flavour" the substrate for these bacteria. This is reflected in decisions to use isomers having a chirality the same as those found in nature. The same applies to the metabolic products released from the bacterial activity and which is less prone to poisoning by naturally-occurring products. These concepts have been successful for the readily-biodegradability of a replacement agent for EDTA. The UK designed and manufactured replacement *S,S'* EDDS degrades to form *l* aspartic acid. Not surprisingly *S,S'*EDDS satisfies the OECD 301E criteria and is being successfully introduced in many areas formally employing EDTA /9,10/.

**Table 1**

OECD tests for ready biodegradability /7/.

DOC = dissolved organic carbon and BOD = biological oxygen demand.

| Test Number | Test Title | Test Parameter | Definitions or Pass Criteria |
|---|---|---|---|
| 301A | DOC Die-away | % DOC Removal | >70% DOC removal within 28 days and within 10-day window after 10% DOC released |
| 301B | Modified Sturm Test | % $CO_2$ Production | >60% of theoretical $CO_2$ production within 28 days and within 10 day window after 10% $CO_2$ has been reached |
| 301C | Modified MITI (I) | % BOD Removal | >60% theoretical BOD removal within 28 days |
| 301D | Closed Bottle | % BOD Removal | >60% theoretical BOD removal within 10 or 14 day window after 10% BOD has been reached |
| 301E | Modified OECD Screening | % DOC Removal | >70% DOC removal within 28 days and within 10 day window after 10% DOC has been released |
| 301F | Manometric Respirometry | % BOD Removal | >60% theoretical BOD removal within 28 days and within 10 day window after 10% BOD has been reached |

The major use of EDTA as a sequestering agent is in the pulp making industry to complex any metal ions present which have a range of nuisance roles but most seriously at the pulp bleaching stages where metal ions cause spotting and a less than white paper product /11/.

Over the last twenty years chlorine based bleaching has given way to more environmental friendly processes, many of them involving oxidation using hydrogen peroxide. These stages are aggravated by the presence of transition metal ions from the wood pulp and from the process water (in practice from large lakes) which permits a Fenton-type reaction and this leads to spotting in the final product /12/.

Traditionally, the largest World usage of EDTA (*circa* 20 000 tonnes) has been to sequester these adventitious metal ions and to produce a clear white product having no spots. Details of the process are given in reference 11. Additional to the clear white paper quality, smoothness of product requires the magnesium to remain in the system in spite of a complexing ligand being introduced to complex iron, manganese, and copper ions – the worst offenders with respect to Fenton reaction initiation. Although paper is produced world-wide, most of the fundamental chemical research occurs in Scandinavia, which means that the amount of ligand(s) introduced has not been pulp factory site specific and so ligand overdosing frequently occurs at other sites /11/.

Realising that doses of EDTA traditionally used had not been optimised, speciation simulation using thousand species models focussed on the salient questions and produced site optimised formulations which (a) fine-tuned the amount of replacement ligand substituted for EDTA (in practise *S,S'*EDDS), and (b) sequestered the maximum amount of problematic transition metal ions whilst leaving the magnesium ions uncomplexed. (c) Previously, EDTA, being such an avid complexer of $Ca^{2+}$, was mainly lost to that metal ion in the feed water (the term used is Calcium Distracted) and only a few percent were remaining for Fenton reaction prevention. The expense of this distraction added to the price of the paper as well as Ca.EDTA posing a serious environmental cost.

By introducing *S,S'*EDDS as a substitute for EDTA there was almost negligible calcium distraction and considerably less agent to go into the environment whence it was readily-biodegradable. This effective 'tuning up' of ligand doses was called "more for less". Further, for some stages of the pulp making process, optimisation employed blends of EDTA and *S,S'*EDDS mixed together in order to achieve some calcium, and total transition metal ion, complexing at a reasonable price and within the degradability limits permitted by OECD regulations. Marketers sometimes advertise their products as "inherently biodegradable" but these do not satisfy the international standard for sustainability of our planet legislated for under their definition of "readily-biodegradable".

Reference 11 reports these achievements in some detail and shows Speciation Efficiency Index and Readily-Biodegradability Index plots understandable by plant managers without their need to master the intricacies of labile equilibria and of complex chemistry.

None of these conclusions ought to be implemented without using pilot scale lab experiments to check that all the data have been verified and the model has been validated for each of the several stages of the pulp making process involving complex chemistry. The price economies of modelling are obvious when one considers the price and environmental cost of doing trial and error dose gradations at pilot plants.

A similar approach has been taken to the control of nutrient metal complexes used in foliar feeding of plants. In agriculture, trace element availability is crucial to achieving higher yields and better crop quality /13,14/. Two pivotal ingredients are ferric, Fe(III), and phosphate ions which, in addition to manganese, copper, molybdenum and zinc deficiencies, cause serious growth problems /15/. Recently foliar application of these agents by spraying onto the leaves of the plants has been shown to be far more efficient than fertilising through soil and roots. Chelating agents such as EDTA are presently added to foliar sprays (i) to prevent metal phosphate/oxide/hydroxide precipitation which blocks the fine spray jets and also (ii) to prevent leaf scorching by free ions.

With the phasing out of EDTA, more environment-friendly alternatives such as *S,S'* EDDS are being introduced but EDTA binds more strongly to Fe(III) than to EDDS; also EDDS is more expensive. Fortunately, it has been possible to use computer simulation modelling of a range of blends of *S,S'*EDDS and EDTA such that precipitation is prevented, environmental readily-biodegradability legality upheld, and purchase cheapness optimised /13/.

Table 2a shows the iron speciation in the presence of EDTA, or EDTA+EDDS, ligand(s) which prevent the iron phosphate precipitate which is potentially jet blocking. However, Table 2b indicates that when *S,S'*EDDS has been used as a total substitute for EDTA, there is a serious precipitate challenge. Hence, a wide range of blends were modelled and the optimum ratios of 60:40 to 70:30 EDDS:EDTA gave essentially the speciation data shown in Table 2a for EDTA alone and also legally satisfies the readily biodegradable criteria of 60% biodegraded within the specified number of days.

**Table 2**

a Speciation percentages of Fe(III) foliar spray system in the presence of excesses of EDTA or the suggested EDDS:EDTA blends when phosphate solid is not formed. b Speciation percentages showing the problem of phosphate precipitate which is present when EDTA is totally replaced with EDDS. Total iron = 15, total chelate(s) = total phosphate = 100 mmol dm$^{-3}$. '–' signifies insignificant amount present.

| | a | a | a | b | b | b |
|---|---|---|---|---|---|---|
| pH | $FeEDTA^-$ | $FeO(OH)_s$ | $FePO_{4\,s}$ | $FeEDDS^-$ | $FeO(OH)_s$ | $FePO_{4\,s}$ |
| 3 | 93 | - | - | - | - | 100 |
| 4 | 98 | - | - | - | - | 100 |
| 5 | 100 | - | - | - | - | 100 |
| 6 | 100 | - | - | - | 97 | - |
| 7 | 100 | - | - | - | 100 | - |
| 8 | 37 | 57 | - | - | 100 | - |
| 9 | 1 | 100 | - | - | 100 | - |

Such an approach to foliar feeding using blending coupled with detailed speciation knowledge has many advantages.-

- The blend range 60:40 through to 70:30 EDDS:EDTA is as effective as the EDTA alone.
- The environmental cost criteria have been satisfied within internationally specified limits.
- The purchase price is cheaper than a straight-forward slot-in replacement of EDDS for EDTA (which would have produced precipitates anyway!).
- Jet blocking both by iron solids and solid $EDDS.H_4$ or a mixture has been prevented /16/.
- Unlike the majority of our speciation studies on biological fluids at the limits of laboratory analysis, the foliar sprays are at easily analysable concentrations and so much data has been aquired to validate our models and confirm that the jet blocking is iron phosphate rather that either oxides, hydroxides, or solid *S,S'*EDDS.
- The ligand supply and agricultural spray formulation industry has confirmed the absence of solids and field trials by the industry are in hand to assess the efficacy of plant growth fertilisation. These are necessarily seasonal and will take more than a year to confirm. Early results are most encouraging /17/.
- The possibility now arises of optimising the foliar supplies of the other micronutrients mentioned above and also of tailoring spray formulations to the particular challenges of a specific plant and soil.

### (b) Speciation data for inorganic agents.

Chemical speciation simulation can contribute to mechanistic knowledge and lead to more accurate risk assessments from exposure to toxic metal ions from the environment. Examples are quoted amongst $^{137}Cs$ considered to absorb from the duodenum in ionic form and from $^{235,238}$ U(VI) weakly absorbed as complex species /18/.

Biological 'intake' becomes 'uptake' when inorganic low molecular mass (lmm) species within the intestine are either ionic, and thus kinetically absorbed through membrane pores involving carrier molecules having ion size specificity and appropriate stereochemistries; alternatively such uptake occurs when net-neutral lmm forms having lipophilicity diffuse into a cell down a species concentration gradient. Models of such as the 'Free Ion Activity Model' – FIAM and the 'Sequential Ligand-ion Interaction Model' –SLIM and have been used to research these respective thermodynamic (FIAM) and kinetic (SLIM) processes /19,20/.

The fluid in the duodenum influences absorption since most inorganic species are in labile equilibrium. The ligands and inorganics in human intestinal fluid, for the purposes of modelling, were assumed to be equal volumes of bile and of pancreatic juices mixed and equilibriated to form duodenal fluid /18/.

The speciation simulation uses computer programs such as JESS to establish mass balance equations of all the species and then equilibrium formation constants for the species being formed from individual components /21-24/. Where exactly matching formation constants are not available, the program extrapolates the likely constant from closely related constants (for example, from a constant measured at a similar temperature or ionic strength). These equations are then solved to indicate the species present at equilibrium.

As previously justified, and validated by several successful models correlated with analytical data on non-radioactive material, most proteinaceous material present was not considered to equilibriate such that it disturbed the order of lmm complexing with the metal ions concerned.

Models were created at $37^0$ and $I = 150$ mmol $dm^{-3}$ to examine how speciation varied with radionuclide concentration ($5x10^{-15}$ up to $10^{-5}$ mol $dm^{-3}$), and with the influence of pH. The reasonableness of the bonding occurring for the most predominant species was checked out /18/.

More than 90% of the caesium present was ionic monovalent $Cs^+$ as previously found by our researches on drinks and for flesh meat.

Above neutral pH, uranium (VI) exists predominantly as $UO_2(CO_3)_5^{8-}$ which places a critical reliance upon the species log K used /25/. However, a detailed analysis of log K values for the other $UO_2(CO_3)_n$ species compared with those of reference 25 showed consistency and so there is confidence that, provided there is sufficient equilibrium time between intestinal contamination (intake) and absorption (uptake), this species is the most important. In order to test the model to its limits exclusion of the uranyl pentacarbonate was modelled and lead to tetracarbonate predominating. The U:C ratio in the tetracarbonate is 1:4 and in the penta is 1:5. This all fits structural data showing that six planar octahedral bonds are subtended at the equatorial $UO_2^{2+}$ and have angles of $60^0$ which accommodates three $CO_3^{2-}$ as four membered chelate rings; the additional carbonate taking the axial or bridging positions.

Reassuringly, neither carbonate complex is relatively low charge density and so they are unlikely to be absorbed (uptaken) through intestinal walls through lipophilic passive diffusion.

Below pH=7, the predominant lmm species is $UO_2H_2(PO_4)_2^{2-}$ which, once again, has a relatively high charge density and four membered uranyl phosphate planar rings. The hydrogens are assumed to be on the non-border phosphate oxygen. Once again, lipophilic absorption is unlikely.

In summary, dose/risk calculations ought to assume that $Cs^+$, alike to $Na^+$ and $K^+$, is extensively absorbed as ionic species whereas $UO_2^{2+}$ in the duodenum is poorly absorbed if at all. (Leggett and Harrison's report of less than 5% is validation) /26/.

Future generations may be challenged by hazards of such metal ions reaching the intestine after geosphere migration from disposal sites. The metal ion acceleration – based upon co-disposed ligands – and retardation – based upon arrest by geological processes such as pores or ion exchange, has been discussed and quantified in risk assessments used in repository planning applications /3/.

Similarly, human uptake from intake of industrial metal ion through wounds has been considered with the use of ligand impregnated wound dressings to prevent the systemic circulation of radionuclides from contaminated wounds /28/.

All of these studies are at parts per billion levels and so beyond the reach of easy *in situ* analysis but, nevertheless, are readily simulated using equilibrium-based models.

### (c) Speciation links between the environment and health.

On the one hand, all of our nutrition and support comes from the environment but, on the other hand, the environment and all industrial chemicals therein are blamed by many for causal links with diseases and other undesirable events. Speciation chemists can help to unscramble these phenomena and to identify real tort.

Some of the public insisting on absolutely safe drugs for therapy, are also de-crying the processes which ensure their safety. Most do not understand the meaning of the word "safe". The UK Chief Medical Officer's annual report for 1995 defined "safe" as a negligible risk being quantified as less than one in one million chance of happening *per annum*, there being no such thing as zero risk. Just as it is incorrect to blame all undesirable events upon chemicals, so too, it would be equally misleading to claim that there are no dangerous chemicals that have been, and that are still being, released upon an unsuspecting public. The checking out of chemicals in our environment began in earnest with the publication of 'Silent Spring' by Rachel Carson in 1962.

For many years, safety cases and licences in the nuclear industry have had to be justified on the grounds of "worst possible case" scenarios. Risk of migration of a hazard (in this case the radioactive element) under the speciation influences of the geochemistry of the site and of any chemicals co-disposed with the radioactive waste material, has been a critical factor in reaching planning and development decisions.

The OECD, the EU, and the UK as members of both, have set up scientific and legislative devices to protect the *status quo* and to ensure that no further damage to the environment and to health arises from chemicals. The global production of chemicals is 400 million tonnes per annum. There are approximately 100,000 chemicals registered in the EU market and which contribute to society's lifestyle as taken for granted today; we encounter about 1000 chemicals daily in everyday activities. It is a gigantic task to assess current, and to back assess retrospectively, hazardous substances. The largest chemical producing region in the World is the EU, producing €1244 billion in 1998 and having a trade surplus of €41 billion /8/.

The perception of this challenge is another formidable obstacle. First, let it be recorded that since the chemical releasing industrial revolution began to produce and to release chemicals some two centuries ago, expected life-spans at birth have doubled from 40 years in 1850 to 80 years today. This paper is not about this perception as the reader is referred elsewhere for improving communications /29/. Rather, this report demonstrates how speciation is pivotal to decisions on continued safe use, to improved risk management, or, in the extreme, to banning an agent. The UK scenario is used as an example but all other countries in the above-mentioned EU, OECD *etc* have taken similar lines and the legislative and policing bodies concerned are currently harmonising their inter-state limits and criteria of concern.

**Table 3**

Persistent organic pollutants targeted by the POPs Convention at World environmental conferences in Stockholm and Johannesburg.

| |
|---|
| Aldrin, Clordane, Dieldrin, Endrin, Heptachlor, Hexachlorobenzene, Mirex, Toxaphene/Camphechlor, Polychlorinated biphenyls, DDT, Dioxins/Furans. |

In common with Europe, a dozen chemical agents have been banned (Table 3). Some 10 000 "high production volume" as defined in EU Existing Chemicals Regulations – *i.e.* they are produced in more than 1000 tonnes per year at least once in the last three years – have been examined from IUCLID data (the European Commission International Uniform Chemical Information Database) concerning Persistence, Bioaccumulation, and Toxicity (PBT) criteria (Table 4). The European Commission's February 2001 White Paper for a Future Chemicals Policy sets out an approach that aims to cover both new, and existing, substances using REACH (Registration, Evaluation, and Authorisation of Chemicals).

**Table 4**

PBT first tier criteria used for guidance of the UK expert committee on hazardous substances.

| |
|---|
| **Persistence, P,** $t_{1/2}$ water > 2 months *or* $t_{1/2}$ soil/sediment > 6 months |
| **Bioaccumulation, B,** log $K_{ow}$ >5 unless Bioconcentration factor (BCF) < 5000 for "greatest concern" *or* >4 and 500, respectively, for "high concern" |
| **Toxicity, T,** Acute lethal effect $L(E)C_{50}$ <1 mg/litre |

Table 5 lists known bio-active agents specifically controlled by existing legislation and which are not included in the categorisations given in Table 4 since strict controls and licencing systems already exist for these agents.

**Table 5**

Hazardous substances already strictly controlled by specific UK legislation.

| |
|---|
| **Pesticides** – the Plant Protection Products Regulations 1995, Control of Pesticides Regulations 1986, and Part III of the Food and Environmental Protection Act 1985. |
| **Biocides** – Biocidal Products Directive 2001 |
| **Food Contact** – Plastics Directive 1998 |
| **Pharmaceuticals and Medicines** – Medicines Act 1968 |
| **Veterinary Medicines** – Medicines Act 1968 |

Table 6 lists web sites for the various bodies involved in setting criteria for UK concern. Essentially, a committee of experts, guided by criteria such as those given in Table 4, gives scientific advice to the UK Chemical Stakeholder Forum. This forum takes advice from bodies having an interest in a chemical *e.g.* the environmentalists, the manufacturing associations, the professional societies, the employee unions, the consumer councils, *etc* and then gives considered advice to Government concerning bans, chemicals likely to cause serious or irreversible damage to the environment, reassurances, import/export controls, the need for new risk management measures, the necessity to generate new data concerning persistence, bio-accumulation and toxicity, the need for research into substitute agents, and means of disposing of wastes and un-used chemicals, *etc.*

This two year old campaign has already resulted in manufacturers accelerating acquisition of safety data, their having such data independently refereed and reported, industry depositing such data with open access bodies charged with responsibility of sharing screening and toxicity data (as part of a larger international effort to reduce animal experiments), and manufacturers initiating research projects to find substitute agents and to optimise (which usually means to reduce) the exposure to the PBT chemicals concerned.

All of these new regulations (a) are being integrated into EU systems which differ marginally between countries and laws, (b) are over and above existing strict regulations for control of known hazards such as radionuclides, pharmaceuticals, *etc* (see Table 5), (c) are reducing the need for some animal experiments, and (d) bring an openness and trust to the perception that chemicals cause all the undesirable aspects of life whereas so-called "natural" or "health" cures currently have the moral high ground. Yet to be achieved is the suppression of cheaply-produced chemical imports from countries not having such stringent environment and health protection regimes in force; the environment is potentially damaged just as much whether the agent is made in a non-EU/OECD country or within those countries having modern environmental sustainability standards. This suggests that the import of cheap non-environmental-friendly manufactured chemicals will, eventually, become history.

Increasingly, chemical speciation data are being used to identify substitutes and to prepare safety strategies. Further details on all these aspects can be read by dialling into the web sites listed in Table 6.

**Table 6**

Lists of web sites describing chemical hazard legislation.

| |
|---|
| http://www.defra.gov.uk/environment/chemicals/ukpolicy.htm |
| http://www.defra.gov.uk/environment/chemicals/csf/criteria.htm |
| http://www.iccahpv.com/hpvchallenge/about.cfm |
| http://www.europa.eu.int/comm/environment/chemicals/index.htm |
| http://www.defra.gov.uk/environment/chemicals/glossary.htm |
| http://www.environment-agency.gov.uk |
| http://www.defra.gov.uk/environment/chemicals/csf/concern/index.htm |
| http://ecb.jrc.it/existing-chemicals/ |
| http://www.defra.gov.uk/environment/chemicals/strategy/03.htm#6 |
| http://www.defra.gov.uk/environment/chemicals/achs/index.htm |
| http://www.food.gov.uk/science/ouradvisors/toxicity/ |
| http://ecb.eu.jrc.it |
| http://ecb.jrc.it/existing-chemicals |
| http://www.europa.eu.int/comm./environment/docum/01262_en.htm |
| http://www.iccahpv.com/ |
| http://www.oecd.org/pdf/M00017000/M00017224.pdf |
| http://www.ospar.org/eng/html/welcome.html |
| http://www.irptc.unep.ch/pops/default.html |
| http:forum.europa.eu.int/Public/irc/env/wfd/library |
| http://www.defra.gov.uk/environment/chemicals/eufuture.htm |
| http://www.cia.org.uk/industry/confidence.htm |
| http:www.iccahpv.com/hpvchallenge/about.cfm |
| http://www.epa.gov/enviro/html/emci/chemref/index.html |
| http://www.coshh-essentials.org.uk |

The strongest link between environment and health is the human diet and chemical speciation factors determine the uptake dependence upon intake. Approximately one third of the food that we eat is good for us, one third has "something" good about it, and the last third is unnecessary. By "diet" we include not only solid materials but also the drinks taken. Both may be a source of nutritional and contaminating trace elements /3/.

In some instances the speciation may have a marked influence upon the uptake and in others the agent may be taken-in unchanged. For example, the absorption of heme-iron from red meat is not significantly influenced by the other ingredients of the whole meal imbibed as it generally enters mucosal cells directly as inert heme complexes/6/. On the other hand, this dietary source differs from that of fruit and vegetable iron in the intestinal tract. Supplements use the second pool.

Slight ligand variations can be critical in the long term.- Many humans use yeast (*saccharomyces cerevisiae*), or its extracts, as rich sources of trace elements but there are wide differences between families of persons and batches of yeast, different families having different bioavailabilities for different elements!

Our grandparents' generation advocated "eat your vegetables, go outside, play, and enjoy yourselves" – this was another way of stating freshly harvested vegetables plus traces of soil impurities (we eat several kilograms per lifetime), exercise, fresh air and relaxing enjoyment; all advice which is even more valuable today. There is much scientific support for these tips. In general, in the absence of illness, therapy, weight irregularities, and food fads, supplements are unnecessary /20,30/.

This is a complex topic that often is dependent upon *ratios* of different elemental species concentrations but the primary consideration must be that competitive labile equilibria and the derived chemical speciation determines the intake/uptake fraction.

### (d) Speciation as a management tool

Powerful programs and computers from Vacca, Linder, May, and Perrin, coupled with the databases of Martell, Smith, Pettit, Powell, and Perrin gave us the possibility of computer modelling of large equilibrium systems /31-35/. Frausto da Silva and Williams used such speciation to explain many bioinorganic and evolutionary phenomena /19/. Taylor, Duffield, and Williams *et al* introduced and expanded the concept of industrial and biological activities being *chemical speciation* dependent rather than *total amount* dependent /6/.

Environmental impact considerations, commencing with Carson, led to readily-biodegradable replacement agents for powerful chelators such as EDTA, the latter persisting in the environment and accelerating metal ion migration /2,36/. In practice, there are no direct "slot-in" ligand replacement solutions because of the different factors which govern the complexing. Rather, all substitute ligand conditions need to be tuned to match the desired speciation /37/.

The fact that all electron donor bio-ligands are pH-dependent in aqueous solution determines that there will be a knock-on effect of having two or more cognate ligands present in the same solution and, usually interacting through common pH-$H^+$-$M^{n+}$ binary complex competition but occasionally, through mixed ligand (ternary) complexes /38/.

This evolution of the subject has now led to the concept of *blending* – the use of two or more ligands being selected from speciation using modelling approaches to optimise desirable complex species, to minimise side reactions, and to be compliant with best metal ion selectivity and environmental objectives as described in section (a) organic ligands under foliar feeding.

Nowadays, the *cost* of a chemical influencing the environment is considered as seriously as purchase and waste disposal *prices*. This is all reinforced by legislation insisting upon the biodegradability of organic species and forbidding enhanced geosphere migration of metal ions in the presence of co-disposed ligands. Often two different complexing tasks are best achieved by two different ligands and the best mixtures of these can only be achieved by simulation rather than trial and error blending which would take an inordinate time for testing. Without speciation simulation, value for research money is unachievable and so a long pilotphase of expensive trial-and-error lab experiments is necessary. With simulation, just a few laboratory experiments are needed to validate the models used.

The communication of such complex issues with decision making management has been facilitated by introducing speciation efficiency indices (SEI) and readily biodegradability indices (RBI) /11/.

This paper has given two, from many, examples from pulp manufacture and foliar feeding of how the chemical speciation optimisation of mixed ligand blends has revolutionised industrial/agricultural/healthcare uses of co-ordination chemistry.

### CONCLUSIONS

Optimistically, management will become more comfortable with purchase and disposal prices and with manufacturing and environmental costs. It has been firmly established that there are no direct 'mole for mole' substitutes in environmental sustainability; rather, chemical speciation knowledge is required to optimise performances and to reduce hazards. Other benefits achieved by introducing speciation simulation are listed in Table 7.

**Table 7**

Some important industrial advantages of speciation knowledge compared with previous approaches.

| |
|---|
| Price advantages |
| Environmental cost benefits |
| Optimised chemical doses (More for less; avoiding distraction) |
| Improved and better-informed management decisions |
| Import controls of non-environmental friendly agents |
| Improvements over conditional constant data |
| Bespoke chelant solutions |
| More objective chemistry for planning applications |
| Chiral specificity benefits |

Improvements in general education ought to include an understanding of hazard, doses and risk relationships so that speciation data be understood, widely accepted, and used to make the best use of all our planet's resources.

### ACKNOWLEDGEMENTS

I am pleased to acknowledge the support of the Speciation Research Group in Cardiff University, Associated Octel in Cheshire, KCL in Helsinki, ADOB in Poznam, and Procter and Gamble Technical Centres in Egham.

**REFERENCES**

1. UK Government strategy statement "Sustainable Production and Use of Chemicals", HMSO, 2001.
2. R. Carson, *Silent Spring*, Penguin, New York, 1962.
3. J. R. Duffield and D. R. Williams, *Chemical Society Reviews*, **15**, 291-307 (1986).
4. L. G. Sillén, *Chemistry in Britain*, 1967; p. 291-297.
5. D. R. Williams, Ed. *An Introduction to Bioinorganic Chemistry*, C. C, Thomas, Publishers, Springfield, Illinois, 1976. ISBN 0-398-03422-2.
6. D. M. Taylor and D. R. Williams, *Trace Element Medicine and Chelation Therapy*, Royal Society of Chemistry, Cambridge, UK, 1995.
7. OECD Report . Testing of Chemicals. Section 3. Degradation and Accumulation. OECD, Paris, 1996
8. Strategy for a Future Chemicals Policy. White Paper, Commission of the European Communities, COM (2001) 88, 27 February 2001. (http://europa.eu.int/comm/environment/chemicals/0188-en.pdf)
9. C. Kezerian and W. Ramsey, US Patent 3158635, November 1964
10. D. Bassett, *Octaquest E – A Biodegradable Chelating Agent*, The Associated Octel Company, Patented 14th November, 1997.
11. P. W. Jones and D. R. Williams, *Inorganica Chimica Acta*, **339**, 41-50 (2002).
12. H. J. H. Fenton, *Proc. Chem. Soc.*, **9**, 113 (1893).
13. J. Davidge, C. P. Thomas, and D. R. Williams, *Chemical Speciation and Bioavailability*, **13**(4), 129-134 (2001).
14. M. D. Bloomberg and E. Y. Lipsitz, Agro Food Industry Hi-Tech, September/October 1999, 18-20.
15. Associated Octel, Ellesmere Port, Technical Bulletin no. 21, 1998.
16. L. Blake, Comparing the Effectiveness of the Ferric Chelates FeEDTA and FeEDDS on Maize and Soyabeans. Report for Rothampstead Plant Laboratory, 1997.
17. T. D. Matthews and D. R. Williams, *Analytica Chimica Acta*, **480**, 119-122 (2003).
18. P. W. Jones, D. M. Taylor, L. M. Webb, and D. R. Williams, *Applied Radiation and Isotopes*, **57**, 159-165 (2002).
19. J. J. R. Frausto da Silva and R. J. P. Williams, *The Biological Chemistry of the Elements*, 2nd Edition, Oxford University Press, 2001.
20. J. Davidge and D. R. Williams, Speciation dependent intake and uptake of essential elements, in: *Metal Ions in Biological Systems*, A. Sigel and H. Sigel (Eds.), Vol 41, Dekker, New York, 2003, in press.
21. P. M. May and K. Murray, *Talanta*, **38**, 1409-1418 (1991).
22. P. M. May and K. Murray, *Talanta*, **38**, 1419-1428 (1991).
23. P. M. May and K. Murray, *Talanta*, **40**, 819-825 (1993).
24. http://jess.murdoch.edu.au/jess/jess-home.htm
25. P. L. Brown and H. Wanner, Predicted Formation Constants using the Unified Theory of Metal Ion Complexation, OECD NEA Report, Nuclear Energy Authority, Paris, 1987.
26. R. W. Leggett and J. D. Harrison, *Health Physics*, **68**, 484-498 (1995).
27. P. W. Jones and D. R. Williams, *Applied Radiation and Isotopes*, **54**, 587-593 (2001).

28. P. W. Jones, D. M. Taylor, M. Finney, A. Iorwerth, D. Webster, K Harding, and D. R. Williams, *Journal of Wound Care*, **10**, 205 – 208 (2001).
29. D. R. Williams, *What is Safe?* The Royal Society of Chemistry, Cambridge, 1998.
30. P. W. Jones and D. R. Williams, The use and role of zinc in wound healing, in: *Metal Ions in Biological Systems*, A. Sigel and H. Sigel (Eds.), Dekker, New York, **41**, 139-183 (2004).
31. P. Gans, A Sabatini, and A. Vacca, *Inorg. Chim. Acta.*, **18**, 237-239 (1976).
32. P. M. May, P. W. Linder and D. R. Williams, *J. Chem. Soc. Dalton*, **1977**, 588-595.
33. R. M. Smith and A. E. Martell, *Critical Stability Constants*, Plenum Press, New York, 1989.
34. D. D. Perrin and B. Dempsey, *Buffers for pH and Metal Ion Control*, Chapman and Hall, London, 1974; pp 176.
35. L. D. Pettit and K. J. Powell, IUPAC Stability Constant Database, www.iupac.org/projects/2000/2000-004-2-500.html, 2000
36. D. R. Williams, *Chemistry in Britain*, 1998; pp. 48-50.
37. J. Davidge and D. R. Williams, *Inorganica Chimica Acta*, 2003, manuscript 005/FDS in press.
38. M. T. Beck and I Nagypal, *Chemistry of Complex Equilibria*, Translation Editor D. R. Williams, Horwood, Chichester, Ed 2, 1990.

